# *Cordyceps militaris* polysaccharides: preparation and topical product application

**DOI:** 10.1186/s40694-023-00150-5

**Published:** 2023-01-25

**Authors:** Mayuree Kanlayavattanakul, Nattaya Lourith

**Affiliations:** 1 School of Cosmetic Science, Mae Fah Luang University, Chiang Rai, 57100 Thailand; 2 Phytocosmetics and Cosmeceuticals Research Group, Mae Fah Luang University, Chiang Rai, Thailand

**Keywords:** *Cordyceps militaris*, Pharmaceutical product, Polysaccharide, Topical product, Skin care

## Abstract

**Background:**

Topical product derived from the fungus *Cordyceps militaris* was explored as a feasible method for an industrial practice.

**Results:**

The mycelium residue of *C. militaris* that was industrial biotechnological produced was extracted with water at different time conditions under ambient temperature, filtered and lyophilized. The extracts were all light to dark brown powder. The 24 h extraction was significantly (p < 0.01) highest in an extractive yield and total polysaccharides content (TPC) (43.33 ± 0.99% and 144.02 ± 2.06 mg glucose/g crude extract). This extract was proved to be stable following an accelerated stability test with the insignificant (p > 0.05) reduction of TPC (4.95 ± 2.23%). Topical product containing the extract were developed. Skin care preparation containing 0.2% extract was exhibited as the appropriated amount giving the stable cream. The developed *C. militaris* polysaccharide cream was confirmed safe and gained more than 70% of the overall preferences examined in 20 female volunteers.

**Conclusions:**

*Cordyceps militaris* mycelium residue is a beneficial source for pharmaceutical products. The *C. militaris* polysaccharides extract was prepared and qualified in terms of active content and stability. The extract was shown to be compatible with the available cosmetic ingredients. The safe and preferred *C. militaris* polysaccharides skin care cosmetics was developed. Accordingly, *C. militaris* polysaccharides skin care cosmetics that meets all the quality characters which are stable, safe, usable and efficient.

## Background

Polysaccharides are composed of multiple saccharides forming a large branched or unbranched chain. These naturally derived polymers are constructed with simple sugar building blocks. They are hydrated in an aqueous environment, thereby creating the gel structure called hydrogel or hydrocolloid. This system, in which water is immobilized by insoluble polymers, consequently can impart a moisturizing effect. In addition, they are excellent in compatibility with the biological tissues and largely meet the consumers’ preferences toward natural products [1]. Moreover, the acidic nature of natural polysaccharides in accordance to the presences of uronic acid is confirmedly confers to their skin hydrating efficacy [1–3]. Of which, the white biotechnology production of polysaccharides is the major source supplied for cosmetic industry [1]. Furthermore, circular bioeconomy awareness is highlighted as the important issue among fast moving consumer goods (FMCG) especially cosmetics of which, bio-based and sustainable products are highly in demand [4].

*Cordyceps militaris* is a special form of mushroom with the fungal fruiting body formed on an insect larva. This fungus has been used extensively as a traditional medicine and dietary supplement, and popularly cooked in several cuisine especially in East Asian countries [5, 6]. This fungus contains many kinds of active components applicable for cosmeceutical uses and one of them is polysaccharide with moisturizing effect [7]*. C. militaris* contains homogeneous polysaccharide, composed of *d*-glucose with a major linkage form of *α-d*-glucose. The side chains were found at 6-*O* positions once in every 8 glucose residues [8]. *C. militaris* is widely grown using a culture of mushroom tissue or mycelium. The fruiting body is harvested for health promotion productions leaving the mycelium as residue. Accordingly, this left-over material from the *C. militaris* production is therefore challenged for its benefitable application in different products. Natural or bio-economy derived ingredients applicable for topical product industries, cosmetic and personal care products. Which, have been sharply rise alongside of the sustainable development. These industrial sectors are therefore the appointed markets, and fitting with sustainability achievement in the value chain of this biotechnological production.

In this context, active pharmaceutical ingredient (API), polysaccharide, valorized from an industrial biotechnological production of *C. militaris* was objectively to be recovered and developed into a topical product. Polysaccharide extraction of the *C. militaris* mycelium residue was undertaken using water at a different time condition. Quality of the extracts in terms of extractive yield and active principle content, total polysaccharides (TPC), were compared in search on the economic feasible exploitation. The appropriate extract was included for stability evaluation, which is one of the important data for development of topical products. Thereafter, it was developed into the stable and microbial contaminant free skin care cosmetics. Furthermore, safety and sensorial evaluations were examined.

## Results

### Preparation of *C. militaris* polysaccharides

**Fig. 1:**
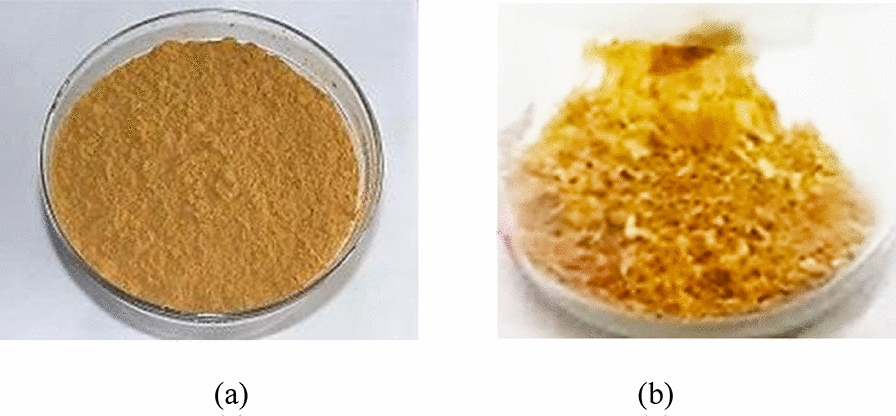
*C. militaris* mycelium (**a**) and its polysaccharide extract (**b**)

The golden-dried powder of *C. militaris* mycelium residue (Fig. 1a) was macerated in water at various time for polysaccharide extraction. All the extracts were light to dark brown powder with a salty odor (Fig. 1b).

**Fig. 2:**
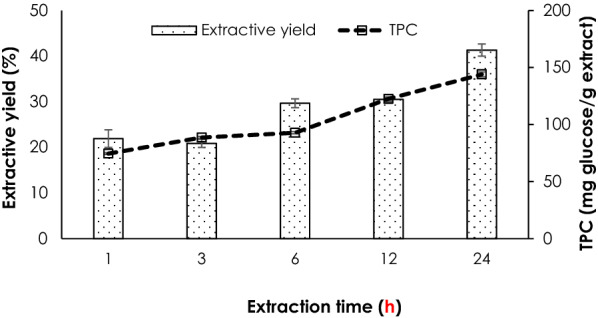
Yield and total polysaccharides content of *C. militaris* polysaccharides extracts

The 24 h extraction time point was significantly (p < 0.01) highest in the extractive yield, which is harmony with the active polysaccharide content, TPC (Fig. 2). This extract was therefore included for further study. *C. militaris* polysaccharide extract was shown to be chemically stable. Which, evidenced by an insignificant reduction of TPC (4.95 ± 2.23%, p > 0.05) following accelerated stability test (TPC_int_ = 144.02 ± 2.06 and TPC_HC_ = 136.91 ± 5.25 mg glucose/g extract). The polysaccharide extract was therefore developed into skin care cosmetics.

### Development of skin care cosmetics containing *C. militaris* polysaccharides

Skin care cosmetics in the form of sleeping mask (or sleeping pack) was developed. This cosmetic is derivatized from night cream, which was used by applying overnight and rinse off in the morning (leave on skin cream mask). The base cream was developed with different ingredients to achieve good cosmetics characteristics as shown in Table 1.

**Table 1:** Development of the stable base creams and creams containing *C. militaris* polysaccharides extract

Ingredient	Formula (% w/w)								
	1	2	3	4	5	6	6A	6B	6C
DI Water									
Sodium EDTA									
Carbopol^®^ Ultrez 21									
Urea	97.50	97.50	96.50	96.50	96.55	96.80	95.00	95.40	95.60
Glycerine									
Propylene glycol									
Carbopol 941									
Dimethicone									
Dimethicone PEG-7 isosterate									
Cyclopentaxyloxane	2.00	2.00	3.00	3.00	3.00	3.40	3.40	3.40	3.40
Dimethicone and Dimethicone crosspolymer									
Phenoxyethanol	0.50	0.50	0.50	0.50	0.70	0.80	0.80	0.80	0.80
Tea tree oil									
Orange oil									
Extract	–	–	–	–	–	–	0.80	0.40	0.20
Triethanolamine	qs to pH 5.5								
Physicochemical property									
Texture	+	+	+ + +	+ + +	+ + +	+ +	+	+ +	+ + +
Color	+	+	+ + +	+ + +	+ + +	+ +	+	+ +	+ + +
Odor	+	+	+ + +	+ + +	+ + +	+ +	+	+ +	+ + +
pH	5.50 ± 0.01	5.52 ± 0.02	5.56 ± 0.02	5.57 ± 0.03	5.62 ± 0.01	5.66 ± 0.03	5.22 ± 0.02	5.65 ± 0.01	5.66 ± 0.01

**Fig. 3:**
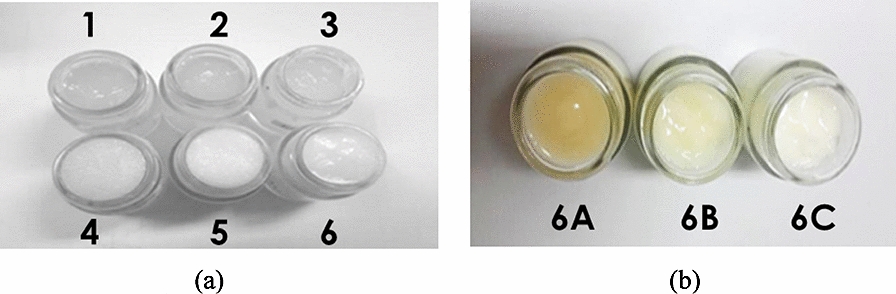
Base creams formula 1–6 (**a**) and creams containing *C. militaris* polysaccharides extract (**b**) 0.8% (6A), 0.4% (6B) and 0.2% (6C)

The base formulas were translucent to opaque white in color (Fig. 3) with a smooth texture and were all remained homogenous following a centrifugation assay.

The base no. 6 with the greatest preferences was therefore chosen for *C. militaris* polysaccharides cream development. Incorporation of *C. militaris* polysaccharides extract acidified the base in regard with the acidic nature of the extract (pH = 4.45 ± 0.01). The base preparation was proven to be compatible with high content of the polysaccharides extract as per all the *C. militaris* polysaccharides formulations were homogenous following the accelerated test by the centrifugation assays. However, the preferences onto texture, color and odor of the 0.4 and 0.8% *C. militaris* polysaccharides formulations were lower than the 0.2% one (Table 1). The 0.2% *C. militaris* polysaccharides cream was therefore included for the accelerated stability test under heat-cool cycles. The preparation was exhibited to be stable (Table 2). In addition, it was confirmed upon the microbial specification as per the base formula.

**Table 2:** Stability assessment of the selected formula under heat-cool cycles

Formula	Initial		Heat-cool cycles	
	pH	Viscosity* (cps)	pH	Viscosity* (cps)
6	5.66 ± 0.03	9956.67 ± 41.66	5.58 ± 0.01	9863.33 ± 35.11
6C	5.66 ± 0.01	6480.00 ± 10.00	5.51 ± 0.01	6333.33 ± 10.66

^*^Spindle no. 6, 100 rpm, torque > 6

### Skin irritation and sensorial evaluations in human volunteers

Safety assessment of the creams was undertaken in the volunteers prior to the sensorial evaluation. Both base and *C. militaris* polysaccharides creams were confirmed as safe, none of the volunteers had any sign of skin irritation in accordance with MII of 0 that equal to that observed skin area exposed with water.

**Fig. 4:**
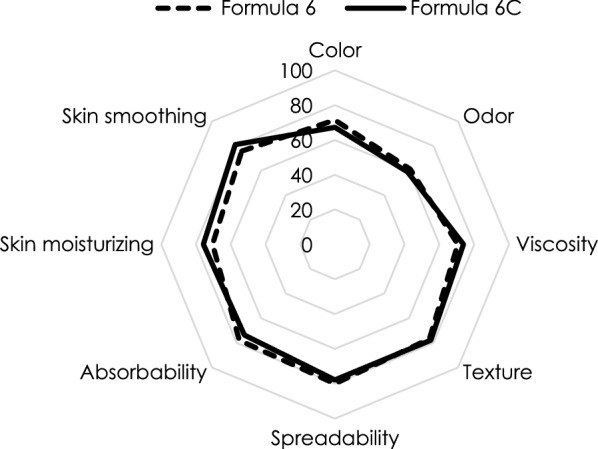
Preference of the developed skin care cosmetics containing *C. militaris *polysaccharides extract

A single-blind, randomized split-face, placebo-control was conducted subsequently. This consumer test was in 20 female volunteers onto the cosmetics’ characters before, during and after use that is also known as the cognitive process simulating the consumers’ attributions. Preference onto the preparation was monitored by the questionnaire by means of check all that apply (CATA) method [9.10]. In addition, this consumer test refers usability and efficacy of the preparation in turn. The preference parameter in terms of appearance and texture of the preparations were comparable (Fig. 4). Overall preferences of both creams were more than 70%, although the *C. militaris* polysaccharides cream was more preferred but insignificant (73.29 ± 3.51% and 73.02 ± 6.08%). It should be noted that the volunteers sensed more skin moisturizing (75.79 ± 4.17% and 70.52 ± 3.51%) and skin smoothing (81.05 ± 5.03% and 75.78 ± 5.65%) than the base cream. Furthermore, none of the volunteers reported any adverse effect following application of the products.

## Discussion

*Cordyceps militaris* has long served as the traditional medicine in several recipes in accordance with its health benefits [5, 6, 8]. Its fruiting body is regarded as the important source of therapeutic polysaccharides [8, 11]. *Cordyceps militaris* polysaccharides are not only important for health promotion but also have their moisturizing potency [7] that co-contribute to anti-aging of skin [11–13]. Cultivation of *C. militaris* is therefore gradually increasing to serve its high demands for several sorts of health promotion products that are increasing year by year [6]. There are the left-over mycelium residue following the fruiting body harvest in turn. In an order to achieve on sustainability thorough the value chain of this medicinal fungus production. The *C. militaris* mycelium residue discarded from the industry was therefore revealed its potency in topical product application. *C. militaris* mycelium polysaccharide was prepared and developed into the safe and preferred skin care product.

The acidic nature of the *C. militaris* polysaccharides extract is in harmony with those of skin hydrating polysaccharides derived from Ceylon spinach [14], malva nut and orchid [2, 3]. *Staphylococcus aureus*, *Escherichia coli*, *Pseudomonas aeruginosa*, *Candida albicans* and *Aspergillus niger* are the prohibited microbes in cosmetics. These microorganisms should be presented in the preparation lower than 10^3^ CFU/g or CFU/ml in term of total colony count, on the basis the standard test methods, i.e., ISO 11930, USP 35, Ph.Er. 7, CTFA M-3 and CTFA M-4 including KoKo test using the test kit. Test kit can monitor bacteria, yeast and fungi that might be contaminated in the preparation if the preservative system is insufficient. The preservation efficacy of the base and *C. militaris* polysaccharides creams were ensured as per both preparations were free from microbial contamination. Safety and sensorial evaluations of the creams were assessed among the volunteers consequently.

Preference onto the preparation was monitored by the questionnaire by means of CATA method [9, 10]. In addition, this consumer test refers to the usability and efficacy of the preparation. Preferences of the developed product were noted by the volunteers.

## Conclusions

*Cordyceps militaris* mycelium residue, which is discarded as the waste following *C. militaris* fruiting body harvested, was evidenced as the beneficial source for API and suitable for pharmaceutical product development. The *C. militaris* polysaccharides extract was prepared and qualified in terms of active content and stability. The extract was found to be compatible with the available cosmetic ingredients. The safe and preferred *C. militaris* polysaccharides skin care cosmetics was developed. Accordingly, the use of *C. militaris* polysaccharides in skin care cosmetics meets all the cosmetics quality characters in terms of stability, safety, usability and efficacy. Valorization of the mycelium wasted from *C. militaris* industrial production is revealed. Sustainable production and consumption perspectives of this white biotechnology with a cleaner production transforming the discarded waste into specialty material supplied for certain, value-added, high-profit industry is exhibited, and fitting with the consumers’ expectation upon circular bioeconomy with an eco-innovation.

## Methods

### Chemical and reagents

The chemical and reagents used for TPC analysis were of analytical grade. Those for cosmetic formulation were of cosmetic grade.

### Preparation of* C. militaris *mycelium polysaccharides

*Cordyceps militaris* mycelium dried powder (World Wide Healthy, Thailand) (5 g) was extracted with DI water (1:10 w/v) with shaking (150 rpm) under ambient condition for 1, 3, 6, 12, and 24 h, separately [14]. The extracting solution was filtrated and lyophilized to dryness. The marc was extracted under the same condition twice more, and the extractive yields were calculated.

### Quality control and standardization of *C. militaris* polysaccharides

#### Total polysaccharides content (TPC)

The active principle content was quality controlled in term of TPC using phenol–sulfuric acid assay, of which glucose was regarded as the standard [3].

#### Stability evaluation

Stability of the selected extract was challenged under heating–cooling cycle (45 ± 2 °C) and (4 ± 2 °C) for 24 h at each temperature for 4 cycles or 8 days. TPC was thereafter re-assessed [3].

### Development of skin care cosmetics containing *C. militaris* polysaccharides

The base formula was firstly developed with different proportions of the ingredients as shown in Table 1. Sensory evaluation during the product development was carried out by an observation of appearance, viscosity and odor, and scored from + to +  +  + (lowest to highest) on the basis of hedonic system [13] by the formulator, in addition to pH (Lab850, Schott, Germany) examination. The preparations were evaluated for stability under accelerated conditions by means of centrifugation at 3500 rpm for 30 min (Micromax, Thermo, USA). The most preferred and stable base was challenged on stability under the accelerated condition similar to that of the extract assessment. Thereafter, it was developed into *C. militaris* polysaccharides creams and re-challenged on stability. pH and viscosity were examined (DV-II^+^Pro, Brookfield, USA). A preservative efficacy test was further undertaken by means of KoKo test using Cult-Dip combi^®^ test (Merck, Poland) and observed following 48 and 72 h incubations.

### Skin irritation and sensorial evaluations in human volunteers

Thai healthy female volunteers aged between 20 and 40 years old were enrolled in the study. This study was approved by the ethical committee of Mae Fah Luang University and undertaken based on the Declaration of Helsinki. Safety assessment of the developed cosmetics (20 µl) was examined by means of a single application closed patch test. Water was used as a negative control. Skin irritation severity was graded 0–4. Observation was undertaking immediately, 24, 48 and 72 h following Finn chamber^®^ (8 mm, SmartPractice, USA) removal. Mean Irritation Index (MII) was calculated. The MII < 0.2 was interpreted as non-irritation [2].

Thereafter, a randomized single-blind, split-face, placebo-control clinical trial was conducted in the same group of the volunteers. The volunteers were directed to apply the base or *C. militaris* polysaccharides creams on either side of their faces on the evening after they had a casual facial cleaned, left overnight and rinsed off in the morning for 7 consecutive days. Preference (%) on the preparations was scored in terms of color, odor, viscosity and texture before use, spreadability and absorbability during use, and skin moisturizing and smoothing after use system [15].

### Statistical analysis

Statistical analysis was performed using the SPSS program for Windows. The significance was set at a reliability of 95%. The result was expressed as the mean ± SD. Assessments in human volunteers were presented as the mean ± SEM.

## Data Availability

The data that support the findings of this study are available from the corresponding author, [NL], upon reasonable request as per the materials.
